# Dr. Francis Mason Hughes

**Published:** 1917-01

**Authors:** 


**Dr. Francis Mason Hughes,** N. Y. University 1877, died at his home on the River Road near Lewiston, Dec. 10, aged 60. He was injured when his auto was struck by a trolley a week before and died of septic pneumonia. His office was at 600 Delaware Avenue, Buffalo.

